# Semaglutide Treatment in Adult-Onset Autoimmune Diabetes: A Case Study With Long-Term Follow-Up and Periodic Evaluation of Beta-Cell Function

**DOI:** 10.7759/cureus.55771

**Published:** 2024-03-08

**Authors:** Andrea Da Porto, Eleonora Varisco, Martina Antonello, Viviana Casarsa, Leonardo A Sechi

**Affiliations:** 1 Department of Medicine, University of Udine, Udine, ITA

**Keywords:** autoimmune diabetes, beta cell function, semaglutide, glp-1 receptor agonists, latent autoimmune diabetes in adults (lada)

## Abstract

Latent autoimmune diabetes of adults (LADA) is a form of autoimmune diabetes that typically occurs in adulthood and has intermediate characteristics between type 1 and type 2 diabetes. To optimize the diagnostic and therapeutic approach, recently, a subclassification of LADA has been proposed based on some clinical features, antibodies, and beta cellular function at onset. In this paper, we expose an interesting case showing the effectiveness of early treatment with a glucagon-like peptide receptor agonist (semaglutide) in maintaining long-term good glycemic control and associated with the preservation of beta-cell function over a five-year observation period in a young woman with LADA.

## Introduction

Type 1 diabetes (T1D) is a condition caused by autoimmune damage of the insulin-producing beta cells of the pancreatic islets, usually leading to severe endogenous insulin deficiency. Although the incidence peaks in puberty and early adulthood, new-onset T1D occurs frequently in adults, and the typical clinical course is characterized by a relatively rapid decline in beta-cell function with subsequent progressive insulin dependence. However, mounting evidence suggests that there are various clinical forms of T1D that are characterized by several aspects including the age of onset, the type of autoantibodies, the speed of loss of cell beta function, pathophysiology, and potential responses to certain drugs [1].

A particular form of diabetes, called latent autoimmune diabetes of adults (LADA), is characterized by relatively slow progression to insulin dependence even in the presence of typical autoimmune patterns. For this reason, adults with new-onset autoimmune diabetes can present mild and nonspecific symptoms due to hyperglycemia and can often be mistaken for type 2 diabetes (T2D) in the early stages. In recent years it has become clear that LADA prevalence is higher than previously thought and may account for 2% to 12% of all cases of diabetes in adults. LADA is often challenging to diagnose and treat due to its atypical age of onset and its heterogeneous clinical presentations. To date, no clear guidelines are available for the management of LADA and the most accepted therapeutic approaches include treatments aimed to preserve residual beta-cell function a mass beyond glycemic control [2].

In 2020, Buzzetti et al. proposed a new subclassification of LADA based on the value of C‐peptide at diagnosis to define different subgroups (T1D endotype 3‐4‐5) of patients that have different disease progression and could benefit from early non-insulinic treatment [1,3]. The new classification was introduced with minor changes in the American Diabetes Association's standards of care treatment in 2022 [4]. However, despite proper classification and treatment, almost 80% of patients with LADA required insulin treatment within five years of diagnosis. In vitro studies have shown that glucagon-like peptide-1 (GLP1) receptor agonists could promote beta-cell preservation; however, in vivo studies on T1D patients are not conclusive [5].

Herein we describe the case of a long-term metabolic response and beta-cell preservation of a young healthy female with LADA (type 1 diabetes endotype 4 (T1DE4)) who was treated early on with a combination therapy including a GLP1 receptor agonist (semaglutide) and metformin.

## Case presentation

A 36-year-old female with a past medical history of autoimmune thyroiditis presented to our clinic with complaints of recurrent genital infections and polyuria. Her pregnancy five years ago was complicated by gestational diabetes diagnosed in the last trimester, requiring treatment with diet for a two-month duration. She had a familiar history of T2D; she conducted a very active lifestyle and played basketball regularly three times a week. Her BMI was 20 kg/m^2^, her waist circumference was 76 cm, and she complained about a recent involuntary reduction in body weight. On the first evaluation, her vital signs and physical exam during the visit revealed no abnormalities. Laboratory investigations were significant for a random blood glucose of 315 mg/dL with a normal anion gap and bicarbonate level. Her HbA1c was 12.3% (111 mmol/l). No blood or urinary ketone was detected. Genetic testing for maturity-onset diabetes of the young (MODY) was negative. Because of her young age, lean body habitus, and healthy lifestyle, we checked autoimmunity and found positive anti-glutamic acid decarboxylase (GAD) antibodies (162 IU/mL), antibodies against intracellular epitopes of the tyrosine-phosphatase 2 (43 IU/L), and negative zinc transporter 8 antibodies (< 10 IU/ml), which unveiled the true diagnosis of adult-onset autoimmune diabetes. At diagnosis fasting C-peptide was preserved at 0.65 ng/ml. She was initially treated with metformin 1000 g BID and 10 units of basal insulin (glargine U300).

In the first five weeks of treatment, the blood glucose levels gradually normalized; however, the patient experienced episodes of fasting hypoglycemia even with very low doses of basal insulin. Therefore, basal insulin was interrupted, and she was switched to semaglutide 0.25 mg/week for four weeks and then titrated to 0.5 mg/week. To evaluate and monitor beta-cell function, once stabilized blood glucose was achieved, the patient underwent 180-minute mixed meal tolerance tests (MMTTs) to evaluate the C-peptide peak at 12, 24, 36, 48, and 60 months after diagnosis. MMTTs were performed using a standardized method in the morning (between 7 and 10 a.m.) following overnight fasting at the in-patient diabetes unit. No coffee, tea, alcohol, chewing gum, or smoking was allowed before the test. The patient ingested 6 mL/kg of the standard liquid meal (BOOST® by Mead-Johnson, LLC, Chicago, USA). Blood samples for glycemia, C-peptide, and insulin levels were taken at mealtime (t0) and time points (in mins) of 30, 60, 90, 120, 150, and 180.

**Figure 1:**
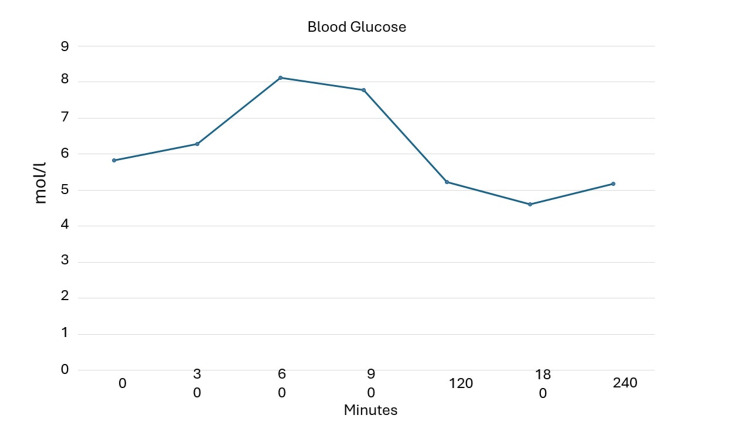
Mixed meal tolerance test at 60 months from diagnosis to assess glucose levels.

**Figure 2:**
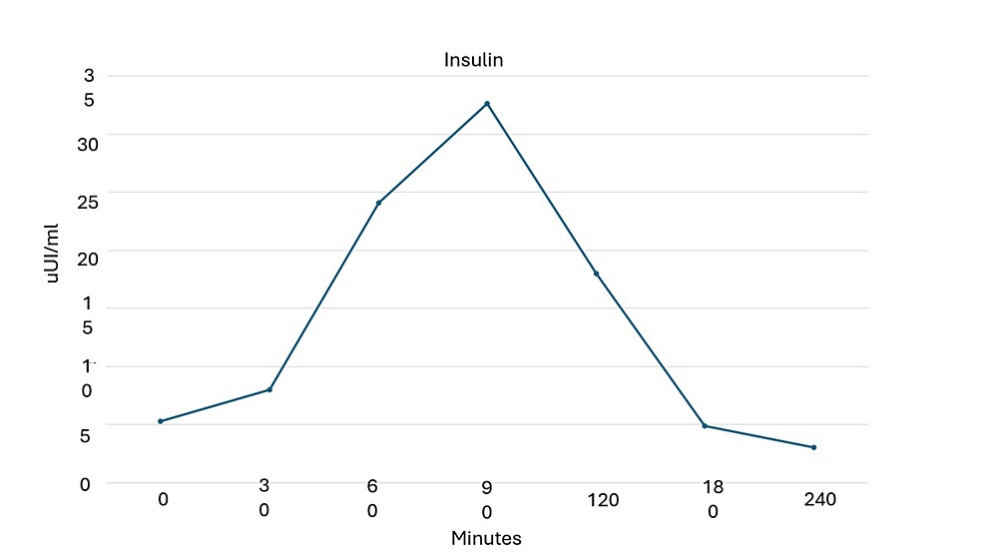
Mixed meal tolerance test at 60 months from diagnosis to assess insulin levels.

**Figure 3:**
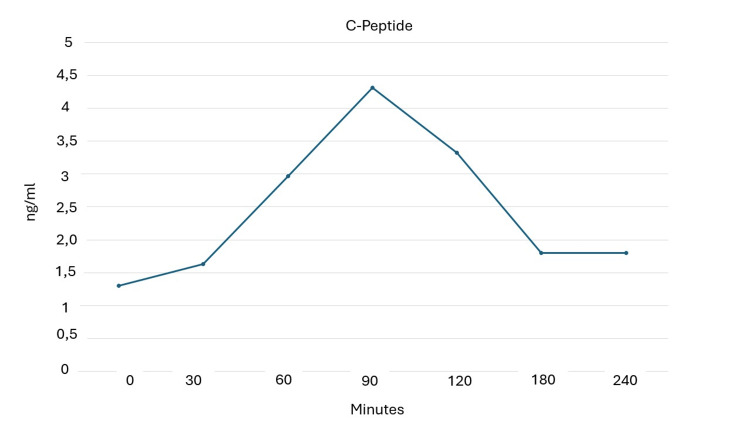
Mixed meal tolerance test at 60 months from diagnosis to assess C-peptide levels.

Trends in anthropometric parameters and HbA1c and beta-cell functional markers are shown in Table 1. During the follow-up period (five years) the patient maintained good glycemic control until 36 months when there was a mild increase in Hb1Ac (7.1%). Semaglutide was titrated to 1 mg/week. The final MMTTs (Figures 1-3) showed a good and preserved beta-cell function up to 60 months after LADA diagnosis.

**Table 1:** The trends in anthropometry, glycemic control, and beta-cell function over the follow-up period DCCT: Diabetes Control and Complications Trial, HOMA: Homeostasis Model Assessment Index, BMI: Body mass index, CV: Coefficient of variation

	12 months	24 months	36 months	48 months	60 months
Fasting Glucose (mmol/)	6.22	6.56	7.5	6.06	5.44
HbA1C (DCCT)	5.9	6.5	7.1	5.4	5.8
Fasting C-Peptide (ng/ml)	0.88	0.91	1.4	0.94	1.3
Fasting Insulin (uMol/L)	3.9	4.3	5.5	4.5	5.3
HOMA Index	1.1	1.3	1.8	1.2	1.3
C-Peptide Peak (ng/ml)	2.5	3.1	4	3.9	4.3
BMI	22.3	21.8	22.1	21.7	21.2
CV	76	76	78	77	74

## Discussion

In this paper, we expose an interesting case showing the effectiveness of early treatment with semaglutide plus metformin in maintaining good glycemic control and associated preservation of beta-cell function over a five-year observation period.

We have seen that HbA1c, fasting glucose, fasting C-peptide, and under stimulus C-peptide peak were stable over the follow-up period, suggesting a potential role of the GLP1 receptor agonist in preserving beta-cell function even in the presence of autoimmunity.

One potential limitation that should be considered is the misclassification of diabetes. It is known that GADA positivity could be found in almost 20% of patients having T2D. Moreover, the presence of more than one autoantibody likely reflects a T1D phenotype and a high risk of early progression to insulin dependence. Clinical and laboratory characteristics of our patients - including young age, BMI <25 kg/m^2^, and presence of two autoantibodies (GADA and IA2) - allow us to classify the patient’s diabetes as “true” autoimmune diabetes: T1DE4, according to the most recent subclassification [1]. It is known that patients classified as T1DE4 have significantly greater residual beta-cell function, therefore preserving their functionality over time appears more feasible [1].

The role of GLP1 in preserving beta-cell function is well known in T2D and growing evidence suggests that GLP1 receptor agonists, alone or in combination with immunomodulators, could be used as a treatment to preserve residual β-cell mass and restore insulin secretion in recently diagnosed T1D [6].

Experimental findings from in vitro models of autoimmune diabetes demonstrate that liraglutide mediates an anti-inflammatory effect that aids in protecting β-cells from the immune-mediated attack that leads to T1D [7].

Sparse clinical data are available on the efficacy of GLP1 receptor agonist treatment in patients with LADA. In a study by Jones et al., treatment with the GLP1 receptor agonists exenatide or liraglutide did not result in improvements in glycemic control in LADA [8]. Conversely, Pozzilli et al. reported that dulaglutide treatment appeared to be equally effective in lowering HbA1c in GADA-positive diabetes in a short-term period (one year of follow-up) [9]. None of these studies provided data upon the preservation of beta-cell function over time. A recent case series with short follow-up published by Paresh Dandona et al. in the *New England Journal of Medicine* showed that semaglutide, given early after the diagnosis of autoimmune diabetes, improved glycemic control and led to progressive elimination of prandial insulin in all patients and basal insulin in most of them; moreover, the authors observed that fasting C-peptide level increased in all the patients after treatment [10]. In our case, we can provide comprehensive data on beta-cell function during all observations.

The strength of this case is that we performed an accurate and regular assessment of beta-cell reserve during a long follow-up period, thus demonstrating that a long-term treatment with semaglutide plus metformin is a viable therapeutic option for early treatment of adult-onset autoimmune diabetes. Well-designed randomized controlled trials are needed to evaluate the potential of an early treatment with GLP1 receptor agonist in patients with LADA.

## Conclusions

In this article, we share the experience of a young woman with autoimmune diabetes (T1DE4) who has preserved good beta-cell function over five years with the contribution of early treatment with semaglutide. Our experience, in line with other recent case reports, suggests that well-designed randomized controlled trials are needed to assess the potential for early treatment with GLP1 receptor agonists in patients with latent autoimmune diabetes in adults.
